# Efficacy of cognitive behavioral therapy for musculoskeletal pain: a systematic review and meta-analysis

**DOI:** 10.3389/fpsyg.2025.1705679

**Published:** 2026-01-20

**Authors:** Xianjun Liu, Wenxue Yuan, Xinman Gao, Ziqi Zhao, Rilang Leng, Yibing Xia

**Affiliations:** 1 School of General Education, Dalian University of Technology, Dalian, China; 2 Department of Physical Education, Southeast University, Nanjing, China; 3 Faculty of Health Sciences and Sports, Macao Polytechnic University, Macao, Macao SAR, China

**Keywords:** cognitive behavioral therapy, functional disability, musculoskeletal pain, pain catastrophizing, systematic review

## Abstract

**Background:**

Chronic musculoskeletal pain is a prevalent condition that causes substantial personal and societal burden, yet the effectiveness of non-pharmacological interventions remains debated. Cognitive behavioral therapy (CBT) and CBT-oriented cognitive-based interventions have been increasingly applied to pain management, but evidence has been fragmented across populations and treatment modalities. This study aimed to provide an updated synthesis of randomized controlled trials (RCTs) evaluating the efficacy of CBT for chronic musculoskeletal pain.

**Methods:**

We conducted a systematic search of PubMed, Web of Science, Scopus, Embase, PsycINFO, and the Cochrane Library. Eligible studies were screened according to predefined criteria, quality was assessed using the Cochrane Risk of Bias 2 tool (ROB 2.0) and statistical analyses were performed in Stata 15.1.

**Results:**

Fourteen high-quality RCTs involving 2,677 patients were included. Meta-analysis indicated that CBT significantly reduced pain catastrophizing (SMD = −0.77, 95% CI: −1.10 to −0.43, *p* < 0.05), pain intensity (SMD = −0.41, 95% CI: −0.62 to −0.20, *p* < 0.05), and functional disability (SMD = −0.20, 95% CI: −0.36 to −0.03, *p* < 0.05). Subgroup analyses of pain intensity further identified a cluster of six studies showing consistent intervention effects (SMD = −0.22, *p* < 0.001), although the specific factors underlying this consistency remain unclear. Functional disability outcomes varied by measurement tool, with significant and homogeneous effects in the RMDQ subgroup (SMD = −0.21, *p* < 0.05) but nonsignificant, heterogeneous results in the ODI subgroup. For catastrophizing, both CBT and pain-coping skills training (PCST) significantly reduced scores (SMD = −0.74 and −0.89, respectively).

**Conclusion:**

These findings suggest that CBT exerts consistent therapeutic benefits for chronic musculoskeletal pain, supporting its role as a reliable clinical non-pharmacological treatment option.

**Systematic review registration:**

This study protocol has been registered with the International Prospective Register of Systematic Reviews (PROSPERO) (registration number: CRD420251073898).

## 1 Introduction

Musculoskeletal conditions affect more than 1.7 billion people worldwide and are one of the leading causes of disability (Williams et al., 2018). Pain is defined by the International Association for the Study of Pain (IASP) as “an unpleasant sensory and emotional experience associated with, or resembling that associated with, actual or potential tissue damage” (Raja et al., 2020). In this review, we focus on chronic musculoskeletal pain, understood as persistent or recurrent pain lasting at least three months that is primarily experienced in the musculoskeletal or spinal region, and conceptualized within a biopsychosocial framework encompassing nociceptive, neuropathic, nociplastic, and mixed mechanisms, including chronic primary musculoskeletal pain presentations such as chronic widespread pain/fibromyalgia (Fitzcharles et al., 2021; Kosek et al., 2021; Treede et al., 2019). In many chronic musculoskeletal conditions, pain intensity and associated disability are only weakly related to identifiable tissue pathology, reflecting the contribution of altered pain processing as well as psychological and social factors (Gerdle et al., 2014). Chronic musculoskeletal pain frequently leads to functional impairment, reduced quality of life, and psychological distress, presenting significant challenges for management. Pharmacological treatments such as non-steroidal anti-inflammatory drugs, opioids and other analgesics remain widely used, but they generally provide only small-to-moderate improvements in pain and function and may be associated with important adverse effects and risks when used long term (Busse et al., 2018; Chou et al., 2017; Hochberg et al., 2016). In contrast, non-pharmacological approaches such as exercise therapy and physical therapy are recommended as core components of chronic pain management and have shown modest but clinically relevant benefits for many patients (Baeske et al., 2024; Mf et al., 2022). Within this broader non-pharmacological framework, psychologically informed interventions such as cognitive behavioral therapy (CBT) have been increasingly adopted as adjuncts to usual care. CBT aims to enhance coping skills by modifying maladaptive behavior, cognition and emotion and by promoting active self-management (Campbell, 2011; McBeth et al., 2012; Sharpe et al., 2001), and standardized CBT-based programs have been developed specifically for chronic pain populations (Steel et al., 2025). Systematic reviews indicate that CBT produces small to moderate improvements in pain, disability, and psychological distress at post-treatment, with benefits in pain-related disability and mood often maintained at 3–12-month follow-up, whereas effects on pain intensity tend to diminish over time (Williams et al., 2012; Williams et al., 2020). As CBT does not involve pharmacological agents, it avoids the risks of drug dependence associated with long-term analgesic use and can also reduce anxiety, depression, and pain catastrophizing (Alcon et al., 2025; de Lira et al., 2025; Feldmeier et al., 2025).

Despite CBT has demonstrated pain relief benefits, some RCTs report its efficacy to be comparable or inferior to other psychological interventions (Cherkin et al., 2016; Sun et al., 2020). Therefore, a comprehensive meta-analysis is needed to consolidate evidence on CBT’s effectiveness. Recent RCTs have shown increased adoption of CBT variants such as Internet CBT (Dear et al., 2021; Lappalainen et al., 2024; O'Moore et al., 2018; Rickardsson et al., 2021) and self-help CBT (Mace et al., 2024) which have expanded CBT’s reach. The theoretical system, meanwhile, has expanded to the “third wave” of CBT, including mindfulness-based cognitive therapy (Bindicsova et al., 2025; Burgess et al., 2024; Pérez-Aranda et al., 2019) and acceptance and commitment therapy (Anwar et al., 2024; Bendelin et al., 2021; Martel et al., 2024), which emphasize psychological flexibility and non-judgmental acceptance of pain (Chen and Li, 2025). Despite the different forms, the studies are all built based on the core mechanism of CBT and are comparable for integrated analysis.

This study aims to review and quantify the efficacy of CBT for chronic musculoskeletal pain, assessing its impact on pain intensity, functional impairment, and pain catastrophizing. It consolidates recent evidence to support evidence-based treatment strategies and clinical applications.

## 2 Methodology

### 2.1 Study design and registration

This review and meta-analysis followed the guidelines for utilizing the Preferred Reporting Items for Systematic Reviews and Meta-Analyses (PRISMA) (Page et al., 2021). Systematic literature search was conducted using six academic databases (see the “Search strategy” section for details), and the results were synthesized using meta-analysis. This study protocol has been registered with the International Prospective Register of Systematic Reviews (PROSPERO) (registration number: CRD420251073898).

### 2.2 Outcomes

The primary outcome of this study was pain intensity, encompassing all subjective pain perceptions assessed using quantitative tools. Secondary outcomes included physical disability and pain catastrophizing.

### 2.3 Search strategy

We conducted a systematic literature search from inception to April, 2025 among six electronical databases: PubMed, Web of Science, Scopus, Embase, PsycINFO, and the Cochrane Library. The search period spanned from inception to 26 April 2025. Keywords were constructed using a combination of MeSH terms and keywords, with search fields covering titles, abstracts, and keywords. To improve the recovery rate, the search strategy for each database was customized in terms of syntax and field adaptation. We did not include gray literature resources, but this was supplemented through reference tracing during subsequent study screening. The detailed strategy is shown in Table 1.

**Table 1:** PubMed search strategy.

Query number	Search details	Results
#1	“Cognitive behavioral therapy”[MeSH terms]	40,868
#2	“behavioral therapies cognitive”[Title/Abstract] OR “behavioral therapy cognitive”[Title/Abstract] OR “cognitive behavioral therapies”[Title/Abstract] OR “therapies cognitive behavioral”[Title/Abstract] OR “therapy cognitive behavioral”[Title/Abstract] OR “cognition therapy”[Title/Abstract] OR ((“Cognition”[MeSH Terms] OR “Cognition”[All Fields] OR “cognitions”[All Fields] OR “Cognitive”[All Fields] OR “cognitively”[All Fields] OR “cognitives”[All Fields]) AND “Therapies”[Title/Abstract]) OR ((“therapeutics”[MeSH Terms] OR “therapeutics”[All Fields] OR “Therapies”[All Fields] OR “Therapy”[MeSH Subheading] OR “Therapy”[All Fields] OR “therapy s”[All Fields] OR “therapys”[All Fields]) AND “Cognition”[Title/Abstract]) OR “therapy cognitive behavior”[Title/Abstract] OR “behavior therapies cognitive”[Title/Abstract] OR “cognitive behavior therapies”[Title/Abstract] OR “therapies cognitive behavior”[Title/Abstract] OR “therapy cognition”[Title/Abstract] OR “behavior therapy cognitive”[Title/Abstract] OR “cognitive behavior therapy”[Title/Abstract] OR “cognitive psychotherapy”[Title/Abstract] OR “cognitive psychotherapies”[Title/Abstract] OR “psychotherapies cognitive”[Title/Abstract] OR “psychotherapy cognitive”[Title/Abstract] OR “therapy cognitive”[Title/Abstract] OR “cognitive therapies”[Title/Abstract] OR “therapies cognitive”[Title/Abstract] OR “cognitive behaviour therapy”[Title/Abstract] OR ((“Behavior”[MeSH Terms] OR “Behavior”[All Fields] OR “Behavioral”[All Fields] OR “behavioural”[All Fields] OR “behavior s”[All Fields] OR “behaviorally”[All Fields] OR “Behaviour”[All Fields] OR “behaviourally”[All Fields] OR “behaviours”[All Fields] OR “behaviors”[All Fields] OR “pattern”[All Fields] OR “pattern s”[All Fields] OR “patternability”[All Fields] OR “patternable”[All Fields] OR “patterned”[All Fields] OR “patterning”[All Fields] OR “patternings”[All Fields] OR “patterns”[All Fields]) AND “therapies cognitive”[Title/Abstract]) OR “behaviour therapy cognitive”[Title/Abstract] OR “cognitive behaviour therapies”[Title/Abstract] OR “therapies cognitive behaviour”[Title/Abstract] OR “therapy cognitive behaviour”[Title/Abstract] OR “cognitive therapy”[Title/Abstract] OR “cognitive restructuring”[Title/Abstract] OR “pain coping skills training”[Title/Abstract] OR “activity pacing”[Title/Abstract] OR “graded exposure”[Title/Abstract] OR “relaxation training”[Title/Abstract] OR “Mindfulness”[Title/Abstract] OR “problem solving therapy”[Title/Abstract]	76,928
#3	#1 OR #2	101,183
#4	“Chronic Pain”[MeSH Terms]	26,872
#5	“pain chronic”[Title/Abstract] OR “widespread chronic pain”[Title/Abstract] OR “chronic pain widespread”[Title/Abstract] OR ((“Pain”[MeSH Terms] OR “Pain”[All Fields]) AND “widespread chronic”[Title/Abstract]) OR “chronic primary pain”[Title/Abstract] OR “pain chronic primary”[Title/Abstract] OR “primary pain chronic”[Title/Abstract] OR “chronic secondary pain”[Title/Abstract] OR “pain chronic secondary”[Title/Abstract] OR “secondary pain chronic”[Title/Abstract] OR “persistent pain”[Title/Abstract] OR “long term pain”[Title/Abstract] OR “musculoskeletal pain”[Title/Abstract] OR “chronic musculoskeletal pain”[Title/Abstract] OR “Fibromyalgia”[Title/Abstract] OR “low back pain”[Title/Abstract] OR “chronic low back pain”[Title/Abstract] OR “neck pain”[Title/Abstract] OR “chronic neck pain”[Title/Abstract] OR “shoulder pain”[Title/Abstract] OR “arthritis pain”[Title/Abstract] OR “Osteoarthritis”[Title/Abstract] OR “rheumatoid arthritis”[Title/Abstract] OR (“Sports-related”[All Fields] AND “chronic pain”[Title/Abstract]) OR “chronic tendinopathy”[Title/Abstract] OR “plantar fasciitis”[Title/Abstract]	312,734
#6	#4 OR #5	331,808
#7	“Randomized controlled trial”[Publication Type]	637,668
#8	“Randomized controlled trial”[Title/Abstract] OR “Randomized”[Title/Abstract] OR “placebo”[Title/Abstract]	887,847
#9	#7 OR #8	1,120,285
#10	#3 AND #6 AND #9	1,228

### 2.4 Study selection and eligibility criteria

Potential studies retrieved were carefully reviewed based on titles and abstracts by two independent reviewers, and duplicate and irrelevant studies were removed, Any disagreements were resolved through discussion or consultation with a third reviewer. Inclusion criteria were pre-established prior to screening and included: ① Inclusion criteria were pre-established prior to screening and included: ① clinical trials evaluating the effects of cognitive behavioral therapy (CBT) or CBT-oriented cognitive-based interventions (e.g., acceptance and commitment therapy, compassion-focused therapy, pain coping skills training) on chronic musculoskeletal pain;② the study design was a randomized controlled trial (RCT), and participants were adults with chronic musculoskeletal pain, defined as pain primarily affecting the musculoskeletal or spinal region and persisting for at least three months, consistent with contemporary IASP classifications of chronic primary and secondary musculoskeletal pain. This definition encompassed nociceptive, neuropathic, nociplastic and mixed pain mechanisms, and included conditions such as non-specific low back pain, osteoarthritis, radicular pain and fibromyalgia, provided that musculoskeletal or spinal pain was the predominant complaint. Studies with the following characteristics were excluded: ① studies that adopted an observational design were excluded ② studies that were review articles (such as systematic reviews, narrative reviews) were excluded, ③ literature that could not be obtained in full text ④ literature materials that were not in English. This study did not set restrictions on participant age, gender, race, or sample size to expand the representativeness and external validity of the included studies. Although small sample studies may introduce bias, we will use sensitivity analysis and other methods to control it in subsequent analyses.

### 2.5 Quality assessment

The risk of bias of the included studies was assessed by two independent reviewers using the Cochrane Risk of Bias tool version 2.0 (ROB 2.0) (Sterne et al., 2019). The assessment was conducted around the primary outcome and secondary outcome indicators, covering five dimensions: ① Bias in the randomization process. ② Bias in intervention deviation. ③ Bias in missing outcome data. ④ Bias in outcome measurement. ⑤ Bias in selective reporting of outcomes. Each item was rated according to the ROB 2.0guidelines (low risk, some concern, or high risk), and any disagreements were resolved by a third researcher. No studies were excluded due to risk of bias, but the potential impact of study quality on the pooled effect size was explored in subgroup analyses. We documented potential sources of heterogeneity, including blinding, control group type, mean participant age, total intervention duration and number of interventions, study country, and CBT intervention modality. Exploratory subgroup analyses were conducted for some outcome variables: physical disability outcomes were grouped and summarized by scale type; pain intensity outcomes were pooled across five studies with no heterogeneity; and pain catastrophizing was compared by subgroup based on intervention modality. These analyses provided support for assessing the robustness of the results and explaining heterogeneity.

### 2.6 Data extraction and management

After completing study screening and bias risk assessment, we used a pre-set Excel spreadsheet to extract study data, including the name of the first author, publication year, study country, sample size, mean age, measurement time point, intervention and control regimen, and data on primary and secondary outcome indicators such as pain intensity, functional disability, and pain catastrophizing. Literature screening, quality assessment, and data extraction were independently completed by two researchers, and disagreements were resolved through discussion.

For some studies that did not provide numerical values but presented results in graphs, we used Engauge Digitizer software to visually estimate the images and used Python programming to read the mean and standard deviation of each time point. The extraction strategy was based on previous studies (Yi et al., 2020), and the values derived from image estimation were annotated in the results section.

In addition, if there were three-arm trials with multiple control groups (such as TAU and waiting groups), in order to avoid repeated inclusion of samples, we merged the control groups according to the recommended method of the Cochrane Manual for Systematic Reviews (Higgins, 2022). The weighted average formula was used for the merged mean:


$$M=(N_{1}M_{1}+N_{2}M_{2})/(N_{1}+N_{2})$$

The pooled standard deviation is:


$$\begin{array}{l} \mathrm{SD}=\\ \surd [(N_{1}–1)\;{{\mathrm{SD}}_{1}}^{2}+(N_{2}–1)\;{{\mathrm{SD}}_{2}}^{2}+(N_{1}N_{2}/(N_{1}+N_{2}))\times {\left(M_{1}–M_{2}\right)}^{2}]\\ /(N_{1}+N_{2}–1) \end{array}$$

### 2.7 Data synthesis

For each outcome, we defined a single primary time point for quantitative synthesis. In each trial, “post-treatment” was defined as the first outcome assessment conducted after completion of the randomised intervention period. When multiple post-randomisation assessments were reported (e.g., end of treatment, 3 months, 6 months), we extracted the measurement closest in time to the final treatment session as the primary endpoint, in order to summarise the immediate effect of the intervention. Follow-up schedules and durations beyond this point (e.g., 3–12 months) varied widely across trials, and only a small number of studies contributed data at any comparable time window. It was therefore not feasible to pre-specify and analyse separate short-, medium- and long-term follow-up categories without substantially reducing statistical power and increasing imprecision. Consequently, our main meta-analyses focus on post-treatment effects, and longer-term outcomes are summarised narratively where reported. The implications of this decision for interpreting the durability of treatment effects are addressed in the Discussion.

All statistical analyses were performed using STATA software (version 15). For each outcome, effect sizes were calculated as standardized mean differences (SMDs) with 95% confidence intervals. The SMDs were interpreted according to conventional thresholds, with absolute values < 0.20 considered trivial, 0.20–0.49 small, 0.50–0.79 moderate, and ≥ 0.80 large (Cohen, 1988). For the primary pain outcome, we included patient-reported measures that contained a dedicated pain domain. Trials most commonly used unidimensional pain scales such as the Numeric Pain Rating Scale (NPRS) or visual analogue scales (VAS). When multidimensional health-status instruments were used (e.g., the Arthritis Impact Measurement Scales-2 (AIMS2), the Impact of Rheumatic Diseases on General Health and Lifestyle (IRGL), or the Western Ontario and McMaster Universities Osteoarthritis Index (WOMAC), we extracted only the pain subscale scores rather than the composite total scores. The pain domains of these instruments are designed to capture the intensity and/or frequency of musculoskeletal pain over a defined recall period, and have been shown to perform comparably to unidimensional pain scales in chronic musculoskeletal conditions (Bjerre-Bastos et al., 2022). On this basis, we treated all extracted pain outcomes as conceptually similar measures of pain intensity, and used standardized mean differences (SMDs) to synthesise results across instruments with different score ranges and formats. We acknowledge, however, that residual differences in instrument content and recall period may contribute to between-study heterogeneity and this is noted in the Discussion when interpreting the pooled pain effect. Although the Pain Catastrophizing Scale (PCS) was consistently used across studies assessing catastrophizing, SMD was adopted to maintain consistency across outcomes that were measured using different instruments (e.g., VAS, NRS, MPQ for pain; ODI and RMDQ for disability) and to account for variations in PCS reporting formats (Luo et al., 2018; Shi et al., 2023). Inter-study heterogeneity was assessed using the I^2^ statistic, where 25, 50, and 75% represented low, moderate, and high heterogeneity, respectively. To explore the sources of heterogeneity, given sufficient data (≥2 studies per subgroup), we conducted exploratory subgroup analyses based on pre-specified categorical variables, including: type of cognitive intervention (traditional cognitive behavioral therapy versus other cognitive interventions such as acceptance and commitment therapy, cognitive-focused therapy, and cognitive behavioral support therapy), type of musculoskeletal disorder (e.g., low back pain versus other musculoskeletal pain), type of outcome measure (e.g., different pain or disability instruments) to examine whether the observed effects were consistent across measurement tools. These subgroup analyses were pre-planned to explore potential effect modifiers and were interpreted as exploratory analyses. Publication bias was assessed using funnel plots and Beg’s test (Viechtbauer, 2007). Sensitivity analysis used the leave-one-out method (Viechtbauer, 2007) to exclude individual studies one by one to assess their impact on the total effect size. In addition, outliers and data based on graphical estimates were excluded to test the robustness of the results.

## 3 Results

### 3.1 Study screening and selection process

**Figure 1:**
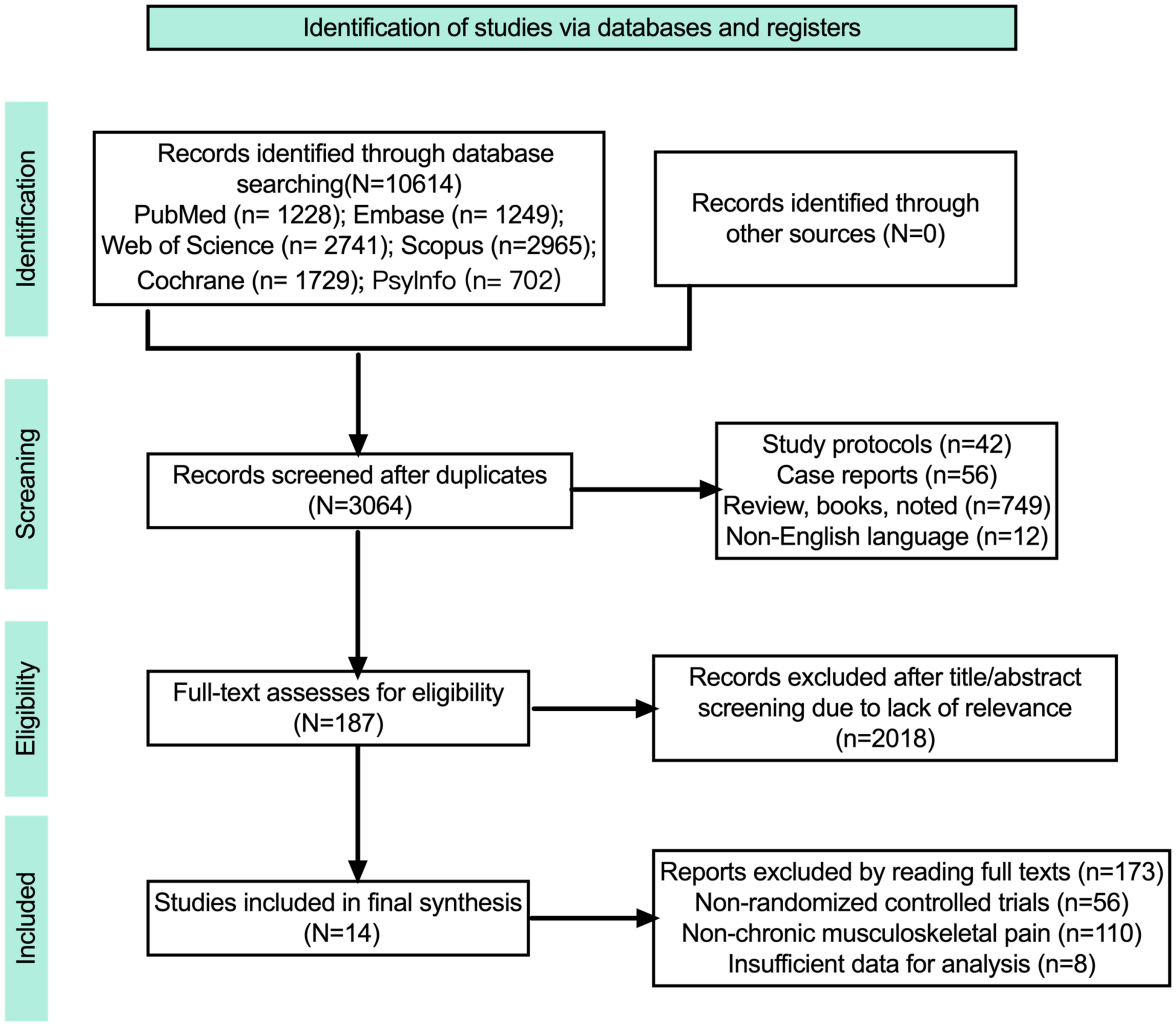
PRISMA flow diagram.

A preliminary search of six academic databases retrieved a total of 10,614 articles: PubMed (*n* = 1,228), Scopus (*n* = 2,965), Embase (*n* = 1,249), PsyInfo (*n* = 702), the Cochrane Library (*n* = 1,729), and Web of Science (*n* = 2,741). Duplicates were removed, and 3,064 articles remained for title and abstract screening. Subsequently, 187 potentially eligible articles were reviewed in full text, ultimately resulting in the inclusion of 14 studies. The literature screening process was independently conducted by two researchers, with any disagreements resolved by a third researcher. The screening process is detailed in the PRISMA flow diagram (Figure 1).

A total of 14 RCTs were included, involving 3,286 patients with chronic musculoskeletal pain, including 1,609 in the intervention group and 1,677 in the control group. Detailed characteristics of each study are summarized in Tables 1, 2. Post-treatment assessments were conducted immediately after the intervention in all trials, and follow-up durations (where reported) ranged from 3 to 12 months Participants were predominantly adults with chronic low back pain, osteoarthritis, rheumatoid arthritis or fibromyalgia; mean ages mostly ranged from the mid-40s to late-60s and women constituted the majority of each sample (Table 2). Interventions comprised traditional CBT and related CBT-based approaches delivered in individual or group formats, and were compared with usual care, education, active physical/medical treatments or wait-list controls (Table 3). Outcome assessment time points ranged from immediate post-treatment to 12-month follow-up, as summarised in Table 3; for the quantitative synthesis, the primary time point was the first post-treatment assessment, as specified in the Methods.

**Table 2:** Summary characteristics of included studies.

First author	Clinical condition	Sample characteristics	Sample (exp/con)	Outcomes
Ng et al. (2015), Australia	LBP	Total *N* = 33 Group CFT: *n* = 18, women NR; age, mean (SD) = 16.3 (1.5) Usual care group: *n* = 15, women NR; age, mean (SD) = 15.2 (1.5)	18/15	Disability (RMDQ); Pain intensity (NPRS); Catastrophizing (PCS)
Andersen et al. (2022), Denmark	Whiplash	Total *N* = 91 (76.9% women); age, mean (SD) = 39.3 (12.1) † V-CBT: *n* = 47, Waitlist: *n* = 44	47/44	Disability (PDI); Pain intensity (NRS); Catastrophizing (PCS)
He and Luo (2025), Brazil	LBP	Total *N* = 80 Group CFT: *n* = 40, women = 60%; age, mean (SD) = 49 (10.59) Core exercises and manual therapy: *n* = 40, women = 50%; age, mean (SD) = 50.45 (10)	40/40	Disability (ODI); Pain intensity (NPRS)
Bennell et al. (2018), Australia	Hip Osteoarthritis	Total *N* = 137 Group CFT: *n* = 67, women = 60%; age, mean (SD) = 49 (10.59) Education: *n* = 70, women = 50%; age, mean (SD) = 50.45 (10)	67/70	Disability (WOMAC); Pain intensity (NRS); Catastrophizing (PCS)
Alda et al. (2011), Spain	fibromyalgia	Total N = 169 Group CBT: *n* = 57, women = 94.7%; age, mean (SD) = 46.35 (6.71) RPT: *n* = 56, women = 92.9%; age, mean (SD) = 47.12 (6.25) TAU: *n* = 55, women = 96.4%; age, mean (SD) = 47.04 (6.53)	57/112	Disability (FIQ); Pain intensity (WOMAC pain subscale); Catastrophizing (PCS)
Castro et al. (2022), Brazil	LBP	Total N = 148 Group CFT: *n* = 74, women = 59.46%; age, mean (SD) = 46.39 (10.62) Core exercises and manual therapy: *n* = 74, women = 63.51%; age, mean (SD) = 40.43 (11.55)	74/74	Disability (ODI); Pain intensity (NPRS)
Darnall et al. (2021), USA	LBP	Total *N* = 179 CBT: *n* = 60, women = 40%; age, mean (SD) = 45.9 (13.1) Empowered relief: *n* = 60 women = 44%; age, mean (SD) = 49.7 (15.0) Health education: *n* = 59 women = 47%; age, mean (SD) = 48.0 (13.2)	60/119	Disability (PROMIS); Pain intensity (NPRS); Catastrophizing (PCS)
Evers et al. (2002), NLD	Rheumatoid arthritis	Total *N* = 59 CBT: *n* = 30, women = 70%; age, mean (SD) = 53.9 (10.3) Usual care: *n* = 29, women = 72%; age, mean (SD) = 53.5 (12.6)	30/29	Disability (IRGL); Pain intensity (IRGL pain subscale)
Godfrey et al. (2020), UK	LBP	Total *N* = 248, women = 59.3%; age, mean (SD) = 47.9 (14.3) † PACT: *n* = 124, Usual care: *n* = 124	124/124	Disability (RMDQ); Pain intensity (IRGL pain subscale)
Lamb et al. (2010), UK	LBP	Total *N* = 701 Group CBT: *n* = 468, women = 61%; age, mean (SD) = 54 (14.9) Advice alone: *n* = 233, women = 59%; age, mean (SD) = 53 (14.6)	468/233	Disability (RMDQ); Pain intensity (MVKD)
Luciano et al. (2014), Spain	Fibromyalgia	Total *N* = 156 Group ACT: *n* = 51, women = 96.1%; age, mean (SD) = 48.88 (5.94) RPT: *n* = 52, women = 98.1%; age, mean (SD) = 47.77 (5.87) WL: *n* = 53, women = 94.3%; age, mean (SD) = 48.28 (5.71)	51/105	Pain intensity (PVAS); Catastrophizing (PCS)
Riddle et al. (2019), USA	Knee Arthroplasty	Total *N* = 402 PCST: *n* = 130, women = 72%; age, mean (SD) = 62.6 (7.9) Usual care: *n* = 137, women = 64%; age, mean (SD) = 62.7 (7.7) Arthritis education: *n* = 135, women = 63%; age, mean (SD) = 64.2 (8.5)	130/272	Disability (WOMAC); Pain intensity (WOMAC pain subscale); Catastrophizing (PCS)
Rini et al. (2015), USA	Osteoarthritis pain	Total *N* = 113 PCST: *n* = 58, women = 79%; age, mean (SD) = 68.52 (7.65) Assessment-only: *n* = 55, women = 82%; age, mean (SD) = 66.67 (11.02)	58/55	Disability (AIMS2 Disability subscale); Pain intensity (AIMS2 pain subscale)
Zgierska et al. (2025), USA	LBP	Total *N* = 770 CBT: *n* = 385, women = 52.2%; age, mean (SD) = 58 (11.7) Mindfulness: *n* = 385, women = 60.5%; age, mean (SD) = 57.6 (11.0)	385/385	Disability (ODI); Pain intensity (PDI)

† Mean age and sex distribution reported for the total sample only.

**Table 3:** CBT and control arm components.

First author	Intervention	Type of control group	Intervention cycle	Outcome assessment time points	Main findings (summary)
Ng et al. (2015)	CFT	Usual care	8-weeks	8 weeks (post-treatment); 12-week follow-up	Compared with standard coaching care, an 8-session CFT program significantly reduced low back pain intensity and functional disability in adolescent male rowers, and these benefits were maintained at 12-week follow-up.
Andersen et al. (2022)	V-CBT	Waitlist	10-session	3-, 6-,9- and 12- month follow-up	V-CBT reduced pain-related disability versus waitlist at 3 months and maintained these benefits at 12 months
He and Luo (2025)	CFT	Core exercises and manual therapy	10.4 Weeks	8 weeks (post-treatment); 22-week follow-up	Compared with core training plus manual therapy (CORE-MT), CFT produced significantly greater reductions in pain and improvements in function in patients with chronic low back pain after spinal surgery at approximately 10 weeks post-treatment, and these between-group differences were maintained at 22-week follow-up.
Bennell et al. (2018)	PCST + Education	Education	8-session	8 weeks (post-treatment); 24- and 52-week follow-up	At the primary endpoint (24 weeks), adding automated online PCST before an exercise program did not improve walking pain or WOMAC physical function compared with online education alone (both groups improved), although the PCST group showed better function and pain coping at 8 weeks and sustained improvements in pain coping through 52 weeks.
Alda et al. (2011)	CBT	RPT and TAU	10–12 weeks	8 weeks (post-treatment); 6-month follow-up	CBT produced significantly greater reductions in pain catastrophizing than relaxation/progressive training (RPT) and treatment as usual (TAU) at post-treatment and 6-month follow-up.
Castro et al. (2022)	CFT	Core exercises and manual therapy	8-session	8 weeks (post-treatment); 6- and 12-month follow-up	In adults with chronic low back pain, CFT yielded a significantly greater short-term reduction in disability than CORE-MT at 8 weeks, but not in pain intensity, and no significant between-group differences were maintained at 6- or 12-month follow-up.
Darnall et al. (2021)	CBT	Empowered relief and education	8-session	1-, 2-, and 3- month post-treatment	CBT produced clinically meaningful improvements and was superior to health education for reducing pain catastrophizing at 3 months post-treatment. A single-session Empowered Relief class achieved non-inferior outcomes to CBT for pain catastrophizing, pain intensity and pain interference, but was inferior to CBT for physical function.
Evers et al. (2002)	CBT	Usual care	10-session	Post-treatment; 6-month follow-up	In psychosocially at-risk patients with relatively early RA, tailored CBT significantly reduced fatigue and depressive symptoms, but did not produce significant between-group improvements in pain, functional disability, or the catastrophizing-related “worrying” subscale.
Godfrey et al. (2020)	PACT	Usual care physical therapy	5 weeks	3 months post-randomization; 12-month follow-up	In adults with chronic low back pain, the PACT intervention produced modestly greater improvements in disability and related outcomes than standard care at 3 months, but no significant between-group differences were observed at 12-month follow-up.
Lamb et al. (2010)	GCBT	Advice alone	6-session	3, 6, and 12 months post-randomization	Group CBT in addition to best-practice advice resulted in significantly greater improvements in disability and pain at 12 months compared with advice alone, with favourable cost-effectiveness and no treatment-related serious adverse events.
Luciano et al. (2014)	GACT	RPT and WL	8-session	Post-treatment; 6-month follow-up	In patients with fibromyalgia, group ACT (GACT) was statistically superior to recommended pharmacotherapy (pregabalin plus duloxetine) and waitlist for improving functional status (FIQ) and most secondary outcomes at post-treatment, with gains largely maintained at 6-month follow-up (mostly medium effect sizes).
Riddle et al. (2019)	PCST	Usual Care and Arthritis Education	8-session	2, 6, and 12 months post-randomization	Among knee arthroplasty patients with moderate-to-high pain catastrophizing, cognitive-behavioral pain-coping skills training did not provide additional benefit over arthritis education or usual care for WOMAC pain at 12 months, and no significant differences were found for secondary outcomes.
Rini et al. (2015)	PCST	Assessment-only	8-session	Midpoint; post-treatment	In hip and knee osteoarthritis, the PCST program showed high adherence and, compared with assessment-only control, produced lower post-treatment pain in women and higher pain-management self-efficacy in both sexes, with smaller improvements in anxiety, pain interference, and affect.
Zgierska et al. (2025)	CBT	Mindfulness	8-session	6- and 12-month follow-up	In opioid-treated chronic low back pain, both 8-week group mindfulness-based therapy (MBT) and CBT led to significant improvements in pain and function and reductions in opioid dose at 6 and 12 months, with no significant between-group differences and MBT non-inferior to CBT on the coprimary outcomes.

### 3.2 Quality assessment of included studies

**Figure 2:**
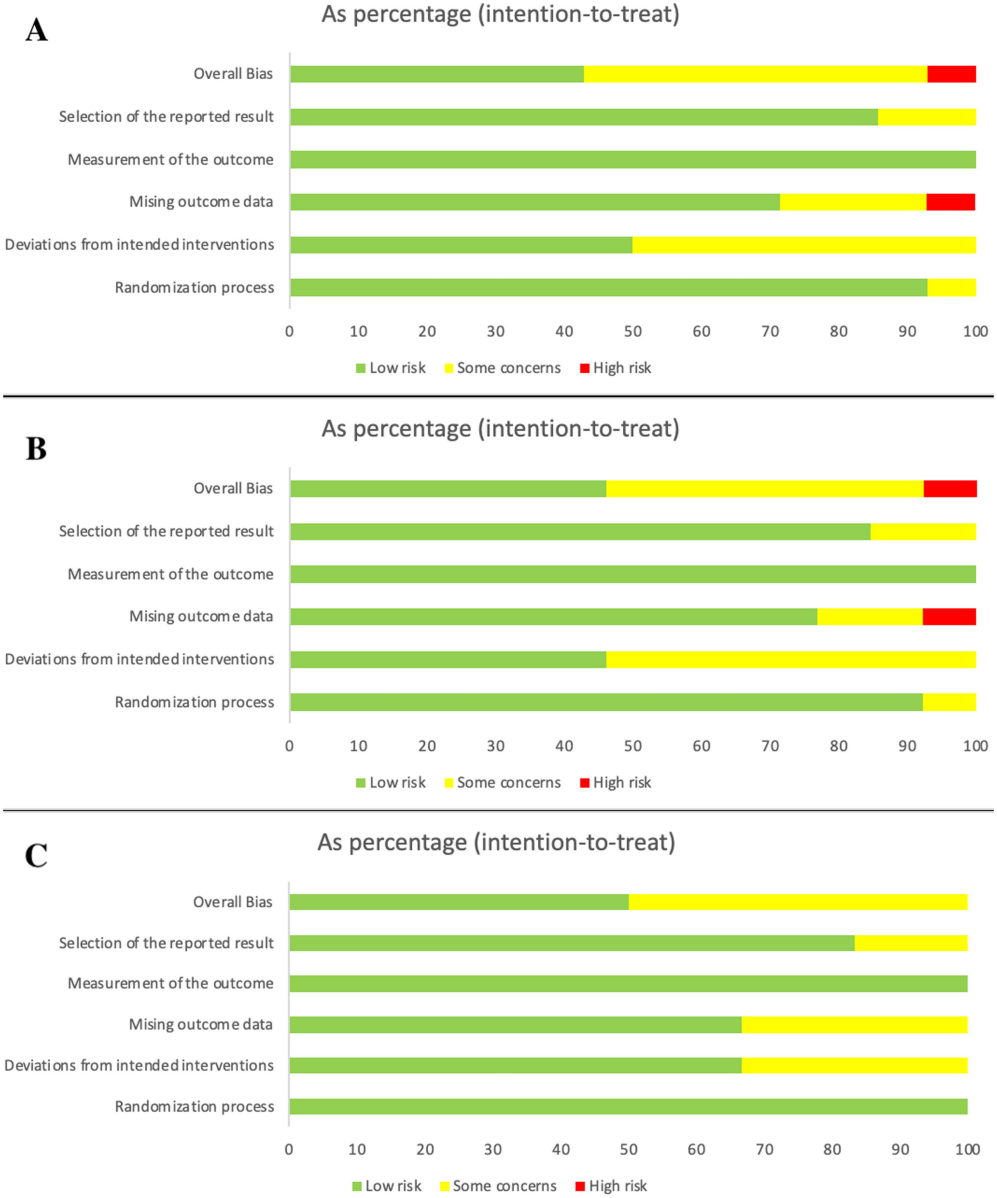
Summary of the results of risk of bias assessment for **(A)** Pain intensity **(B)** Disability function **(C)** Pain catastrophizing.

We used the ROB 2 0.0 tool to assess the risk of bias of the 14 included studies, covering five domains of bias under the intention-to-treat analysis. The results are shown in Figure 2. Regarding “randomization process,” 92.9% of the studies were assessed as having a low risk of bias, with only one study (7.1%) being assessed as having a moderate risk of bias due to an unclear description of the random sequence generation or allocation concealment methods. Regarding “intervention deviation,” 50% of the studies were assessed as having a low risk of bias, while the other 50% were assessed as having a moderate risk of bias, primarily due to poor adherence or missing information during intervention implementation. Regarding “missing outcome data,” 71.4% of the studies were assessed as having a low risk of bias, 21.4% as having a moderate risk of bias, and another 7.1% as having a high risk of bias, possibly due to high rates of loss to follow-up and inadequate missing data handling methods. Regarding “outcome measurement,” all studies were assessed as having a low risk of bias. In terms of “reporting selectivity,” 85.7% of studies were rated as low risk, 14.3% were rated as having some risk of bias, mostly due to lack of registration or incomplete registration information. 42.9% of studies were rated as low risk, 50% were rated as having some risk of bias, and only one study (7.1%) was rated as having a high risk of bias (Figure 2).

14 terms of overall risk of bias, 42.9% of the studies were assessed as having a low risk of bias, while 50% were rated as having moderate risk, and 7.1% were rated as having high risk, mainly due to issues with missing data. Overall, the risk of bias in these studies was considered moderate, suggesting that the evidence quality is moderate for the outcomes assessed.

### 3.3 Meta analyses

#### 3.3.1 Meta-analysis of primary outcomes

##### 3.3.1.1 Pain intensity

**Figure 3:**
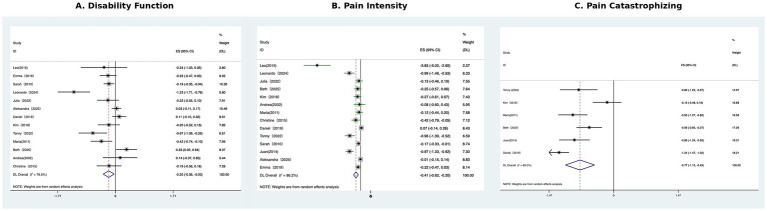
Forest plots of the effects of CBT on **(A)** Disability function **(B)** Pain intensity **(C)** Pain catastrophizing.

This meta-analysis included 14 studies evaluating the effectiveness of CBT-based interventions on pain intensity in musculoskeletal disorders, with a total sample size of 3,286 participants. The most commonly used measurement instrument was the Numeric Pain Rating Scale (NPRS), with other commonly used instruments including the Impact on Rheumatic Diseases of the Groningen List (IRGL), Pain Visual Analog Scale (PVAS), Arthritis Impact Measurement Scale-2 (AIMS2), and Western Ontario and McMaster Universities Osteoarthritis Index (WOMAC). From the forest plot, considerable heterogeneity was detected (I^2^ = 86.2%). Therefore, the random-effects model was performed for the meta-analysis of pain intensity. The results showed that CBT-based interventions significantly reduced pain intensity immediately post-treatment, with an overall effect size of SMD = −0.41, 95% CI: −0.62 to −0.20, *p* < 0.05, this pooled SMD should be interpreted as an overall estimate of patient-reported pain intensity across these different validated instruments, rather than as a value specific to any single scale. This result suggests that CBT has a small-to-moderate effect size advantage in alleviating musculoskeletal pain (Figure 3). Sensitivity analysis showed that individual studies had no impact on the overall effect estimate (Appendix Figure 1).

**Figure 4:**
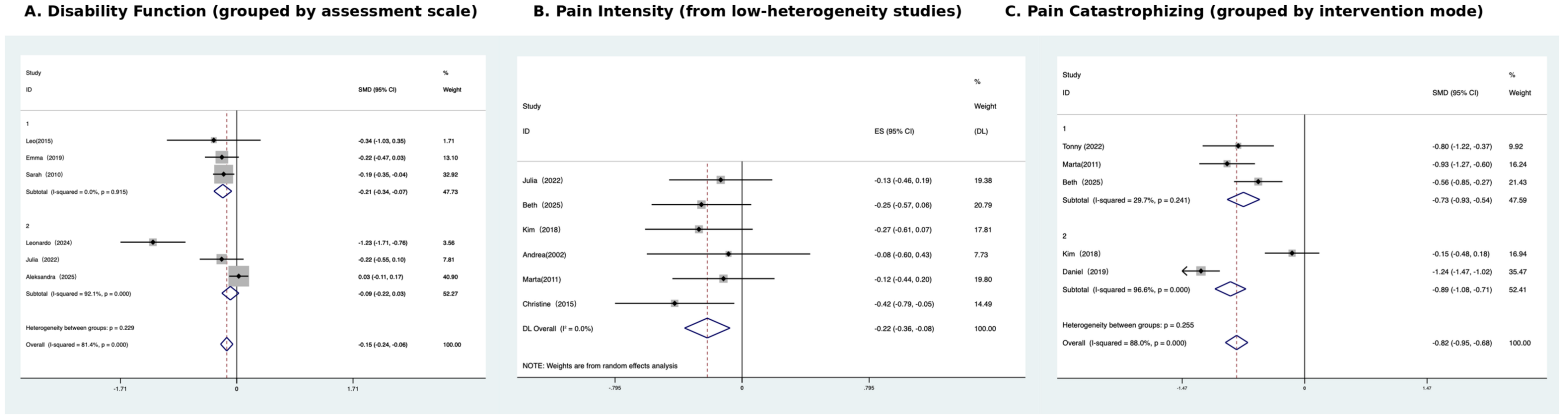
Subgroup Forest plots of CBT effects on **(A)** Disability function (grouped by assessment) **(B)** Pain intensity (from low-heterogeneity studies) **(C)** Pain catastrophizing (grouped by intervention mode).

To further explore potential sources of inter-study effect variation, we conducted subgroup analyses. Although meta-regression failed to identify significant moderating variables, we found that a specific subgroup of six studies exhibited consistent effects after pooling, with a statistically significant effect size of SMD = −0.22, 95% CI: −0.36 to −0.08, *p* < 0.001, indicating high consistency of findings within this subgroup. The subgroups may share commonalities in intervention format, participant type, or study quality (e.g., longer intervention duration, individualized CBT, larger sample size, or lower risk of bias), contributing to these consistent intervention effects. While the specific factors driving this consistency remain unclear, this suggests that this type of study design could serve as a standard or reference in the future (Figure 4).

To assess whether the included studies were subject to publication bias regarding pain intensity, we used Egger’s linear regression test and Begg’s correlation test. The results showed that the Egger’s test was significant (bias = −4.55, *p* = 0.002), suggesting funnel plot asymmetry; the Begg’s test was also significant (z = −3.01, *p* = 0.003), further supporting the possibility of publication bias.

**Figure 5:**
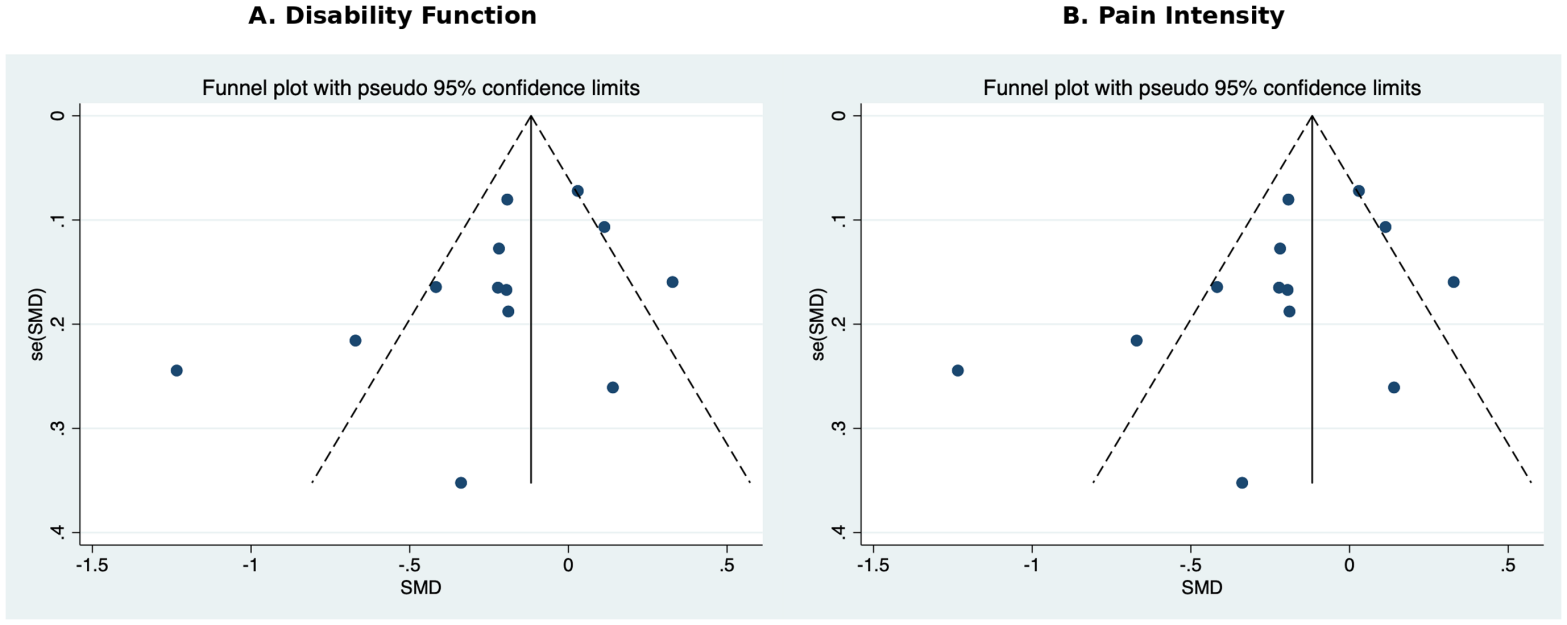
Funnel plots assessing publication bias on **(A)** Disability function **(B)** Pain intensity.

Combined with the funnel plot visualization results, smaller sample size studies tended to report more significant negative effects, suggesting a higher likelihood of positive publication bias due to small sample sizes (Figure 5).

The Duval and Tweedie trim-and-fill method was then used for bias correction analysis. The trim-and-fill algorithm identified no studies for removal or inclusion (0 studies in both cases) under either the fixed-effect or random-effect models, and the pooled effect size remained unchanged, SMD = −0.408, 95% CI: −0.618 to −0.199. This may indicate that, despite statistical testing suggesting potential bias, the current data were not sufficiently skewed to trigger an automatic correction mechanism under the trim-and-fill algorithm.

Thus, although no adjustment was performed using the trim-and-fill method, the Egger and Begg test results suggest that the possibility of publication bias should be considered when interpreting the pooled effect of pain intensity.

#### 3.3.2 Meta-analysis of secondary outcome

##### 3.3.2.1 Disability function

A total of 13 studies were included in the Disability Function meta-analysis, involving a total of 3,137 participants. The most commonly used measurement instrument was the Roland–Morris Disability Questionnaire (RMDQ), with other commonly used instruments including the Oswestry Disability Index (ODI), WOMAC, and Pain Disability Index (PDI). From the forest plot, substantial heterogeneity was detected (I^2^ = 76.5%). Therefore, the random-effects model was performed for the meta-analysis of disability function. The results showed that CBT-based interventions had a small but statistically significant effect on chronic musculoskeletal pain at the end of treatment (SMD = −0.20, 95% CI: −0.36 to −0.03), *p* < 0.05 indicating that CBT has a generally positive effect across studies (Figure 3). Sensitivity analysis showed that individual studies had no impact on the overall effect estimate (Appendix Figure 1).

To further explore the sources of heterogeneity and enhance the interpretability of the meta-analysis results, this study conducted subgroup analyses based on the type of functional disability rating scale used. RMDQ and ODI groups were constructed, each containing three studies. Subgroup analyses revealed strong heterogeneity between the two groups, reaching high levels of heterogeneity. Within the RMDQ scale, there was no heterogeneity (I^2^ = 0.0%, *p* = 0.915), indicating consistency in the findings. The pooled effect size in the fixed-effect model was SMD = −0.205, 95% CI: −0.336 to −0.074, which was significant (z = 3.07, *p* < 0.05), indicating that the CBT intervention based on the RMDQ scale had a statistically significant small effect in improving functional disability, and the results were relatively robust. The pooled effect size for the ODI scale was SMD = −0.094, 95% CI: −0.219 to 0.031, *p* = 0.140, with high heterogeneity (I^2^ = 92.1%, *p* = 0.000), indicating significant differences in results within this subgroup and that the overall effect did not reach statistical significance (Figure 4).

To assess whether this study was subject to publication bias, we created a funnel plot (Figure 5) and performed the Begg test and Egger linear regression test (Appendix Figure 1). The funnel plot showed that the distribution of included studies was relatively symmetrical, with no significant skewness observed. The z-value of the Begg test was −0.85 (*p* = 0.393), and the bias of the Egger test was −2.08, 95% CI: −4.88 to 0.72, *p* = 0.130, neither of which reached statistical significance. Therefore, no evidence of significant publication bias was found in this meta-analysis.

##### 3.3.2.2 Catastrophizing

*T*his study included six articles with a total sample size of 1,178 participants. Pain catastrophizing was assessed using the Pain Catastrophizing Scale (PCS) in all included studies. After testing for heterogeneity, I^2^ = 85% > 50%, indicating strong heterogeneity among the selected articles. Therefore, a random-effects model was used for meta-analysis. The results showed that CBT-based interventions significantly reduced pain catastrophizing, as measured by the Pain Catastrophizing Scale (PCS), at post-treatment (SMD = −0.77, 95% CI: −1.10 to −0.43, *p* < 0.05), which was statistically significant. CBT-based interventions have a moderate-to-large effect on reducing pain catastrophizing (Figure 3). Sensitivity analysis showed that individual studies had no impact on the overall effect estimate (Appendix Figure 1).

To further explore the differences in the effectiveness of different psychological interventions for pain relief, we conducted subgroup analyses based on intervention type. For Pain catastrophizing, at least two studies were available for CBT and PCST, so these were analysed as separate subgroups. One additional trial used Compassion-Focused Therapy (CFT); because only a single CFT study reported PCS, it was not analysed as a separate subgroup but was included in the overall pooled analysis and described narratively. The CBT group included three studies: Andersen et al. (2022), Alda et al. (2011), and Darnall et al. (2021). The combined effect size was SMD = −0.735, 95% CI: −0.930 to −0.540, which was statistically significant (*p* < 0.001). The intra-group heterogeneity was low (I^2^ = 29.7%, *p* = 0.241), indicating that the intervention had a moderate effect on improving pain intensity and the results were stable. The PCST group included two studies: Bennell et al. (2018) and Riddle et al. (2019). The combined effect size was SMD = −0.892, 95% CI: −1.056 to −0.728, which was also significant (*p* < 0.001), but there was high heterogeneity (I^2^ = 96.6%, *p* < 0.001), and caution was needed regarding the interpretability of the results. There was no statistically significant difference between the two groups (*p* = 0.227), indicating that the two interventions had similar overall pain relief effects (Figure 4).

## 4 Discussion

The participants had a variety of pain conditions, such as low back pain, fibromyalgia, and joint pain. CBT was the most common treatment option, with a total of 6 trials. In addition, there were three PCST trials, three CFT trials, and one ACT intervention trial.

This study observed different degrees of intervention effects in the three main outcome indicators, showing a certain hierarchical distribution: CBT-based interventions had the largest intervention effect on pain catastrophizing (SMD = −0.77), followed by pain intensity (SMD = −0.41), while the effect on functional impairment was relatively small (SMD = −0.20). This difference in effect can be explained by the intervention mechanism. Pain catastrophizing is essentially a cognitive bias, manifested as excessive worry about pain, helplessness, and negative expectations, and is the direct target of CBT intervention (Castaño-Asins et al., 2025). CBT can produce significant cognitive changes in a relatively short period of time by reconstructing individuals’ negative cognition of pain, so the intervention effect on this outcome is the most significant. Pain intensity is a composite result of subjective perception and neuro-emotional response. Though partially affected by cognitive regulation, it is still embedded in pathological mechanisms and physical conditions at a deeper level. Therefore, it is reasonable to show a moderate effect at the end of the treatment. Functional impairment indicators cover multiple dimensions such as daily activities, social functions and physical motor ability. Its improvement not only depends on pain relief, but requires the coordinated improvement of cognition, behavior and physical function, which leads to lagged change (Pak et al., 2023). Therefore, the intervention effect of CBT-based interventions on different outcome indicators shows mechanism-related differential characteristics.

This study found that cognitive-based interventions grounded in CBT principles (including CBT, PCST, CFT and ACT) produced a small-to-moderate overall effect on pain intensity in chronic musculoskeletal pain, but the pooled estimate was accompanied by substantial heterogeneity (I^2^ = 86.2%), and therefore should be interpreted with caution. Therefore, we further screened 6 studies with zero heterogeneity (I^2^ = 0.0%) to form a specific subgroup. After merging, a statistically significant positive effect was still retained, SMD = −0.22, 95% CI: −0.36 to −0.08. Compared with the overall analysis, this subgroup is more consistent, which is related to the use of consistent assessment scales, more standardized intervention periods, or more homogeneous sample characteristics in these studies. Although no clear source of heterogeneity was identified in the subgroup with zero heterogeneity, we suspect that moderating factors such as CBT modality, duration of intervention, pain severity, and psychological characteristics of participants may have influenced the variability in the overall effect (Turner et al., 2007). For example, studies with longer interventions, more structured protocols, or greater sample consistency may show more stable outcomes. Future research should explore these potential moderating factors more thoroughly to understand their impact on the efficacy of CBT for pain intensity. Moreover, given the subjective nature of pain measurement, future studies should combine objective pain scales with self-reported measures to provide a more accurate assessment of treatment effects (Yang et al., 2022). The consistent use of validated pain scales across studies could help improve the reliability of findings and reduce bias. In clinical practice, self-reported pain is inherently subjective and can be influenced by psychological factors such as mood, catastrophizing, and expectations (McCahon et al., 2005). These factors may distort the true therapeutic effect of CBT and lead to either an overestimation or underestimation of its efficacy (Crombez et al., 2023). Therefore, clinicians should interpret the results with caution, especially in the presence of subjective reporting biases. Future studies should aim to minimize these biases by incorporating objective pain assessments and considering the psychological profiles of participants. This approach could enhance the robustness of future trials and provide more reliable evidence on the effectiveness of CBT for pain management. This is basically consistent with other meta-analysis studies on chronic pain in recent years. A study integrating 20 randomized controlled trials showed that CBT had a small advantage in relieving pain compared with conventional treatment, SMD = −0.28, 95% CI: −0.39 to −0.16, and the heterogeneity between studies was at a moderate level (I^2^ = 53%) (Rosser et al., 2023). CBT has a certain stability in the intervention effect of relieving the intensity of chronic musculoskeletal pain.

Despite the heterogeneity in the overall effect, the subset of studies with higher methodological consistency showed more stable and consistent positive effects, these results are broadly consistent with previous systematic reviews and meta-analyses of CBT and related psychological therapies for chronic pain. For example, a large Cochrane review by Williams et al. reported small beneficial effects of CBT on pain, disability and distress compared with treatment as usual or active control across a range of chronic non-cancer pain conditions (Williams et al., 2020), while Yang et al. found small-to-moderate improvements in pain and disability in patients with chronic low back pain receiving CBT (Yang et al., 2022). Together with our findings in chronic musculoskeletal pain, this suggests that CBT-oriented cognitive-based interventions have relatively stable but modest effects on pain intensity and disability. CBT-based interventions only showed a mild effect in improving functional impairment, but the heterogeneity was too high, and the scale type obviously constituted a key moderating variable. As a tool specifically used to assess low back functional limitations, RMDQ has a relatively centralized structure and high sensitivity, and the combined results showed no heterogeneity. The ODI scale has a wider dimension and a more complex structure (Chiarotto et al., 2016). The measurement results are more easily affected by individual cognitive understanding or differences in life background, which may lead to greater fluctuations in the within-group effect. This difference in the measurement tool itself may explain the inconsistency in the stability of the results between subgroups. Beyond the subgroup analysis, potential moderating factors could contribute to the observed heterogeneity in functional impairment outcomes. These factors may include variations in CBT modality, intervention duration, patient characteristics (such as age, comorbidities, and baseline function), and pain severity (McCracken and Vowles, 2014). In our article, exploratory analyses did not demonstrate a clear duration–response pattern; however, previous work has suggested that programmes with sufficient intensity and individualisation may be more likely to yield sustained functional improvements in some chronic pain populations (DeBar et al., 2025). These potential moderators should be examined prospectively in future trials to clarify how dose, format and patient characteristics influence the effectiveness of CBT-based interventions and to optimise intervention strategies. Furthermore, the trajectory of improvement in functional impairment is typically more complex than changes in pain intensity. It depends not only on pain relief itself, but also on the restoration of motor behavior, the recovery of daily activities, and enhancements in self-efficacy. Consistent with this distinction, in our analyses disability outcomes were derived from dedicated functional scales, whereas pain outcomes were based on pain-specific subscales of the respective instruments. It is difficult to achieve a complete reversal by psychological intervention alone. This is also reflected in previous digital CBT studies on OA patients (Zhu et al., 2025). Therefore, supporting the improvement of functional impairment requires a more integrated, multi-disciplinary approach, combining CBT with physical therapy, exercise interventions, and behavioral strategies to address the complex nature of functional recovery (Cheng and Cheng, 2019; Scascighini et al., 2008).

Across the outcomes examined in this review, CBT-oriented cognitive-based interventions produced their largest effects on pain catastrophizing, whereas the effects on pain intensity and functional impairment were smaller. This pattern is consistent with the primary cognitive target of these interventions. Pain catastrophizing refers to an individual’s negative cognitive processing of pain, typically characterised by exaggeration, helplessness and persistent attention to pain, and represents a core cognitive construct within CBT theory. In this study, both traditional CBT and related CBT-based interventions demonstrated consistent improvements in catastrophizing, supporting the stability and broad applicability of this theoretical pathway. The relatively large effect size may also reflect the strong structural validity and measurement sensitivity of the PCS, which can detect meaningful changes in pain-related cognitions over relatively short intervention periods (Hayashi et al., 2022). In the subgroup analysis, traditional CBT and its variants showed consistent effect directions in reducing catastrophizing. Although there was a high degree of heterogeneity in the variant CBT, the effect size was significant, indicating that different intervention forms are consistent in the core mechanism of intervention. The reduction of pain catastrophizing observed in this study has significant clinical implications. As pain catastrophizing is a key factor that can intensify pain perception and hinder recovery, addressing it through CBT can significantly enhance treatment outcomes (Quartana et al., 2009). By targeting cognitive distortions, CBT helps individuals reframe their negative thoughts about pain, thereby reducing its emotional and psychological impact (Scarone et al., 2020). This intervention is crucial for patients suffering from chronic musculoskeletal pain, particularly those with high levels of catastrophizing, as it can improve their ability to cope with pain, reduce emotional distress, and prevent the development of more severe pain-related disabilities. Therefore, CBT should be considered a core component of pain management strategies in clinical practice, particularly in treating patients with cognitive distortions related to pain.

In summary, this systematic review and meta-analysis indicates that CBT-oriented cognitive-based interventions for chronic musculoskeletal pain provide small-to-moderate improvements in pain intensity and psychological outcomes, and small improvements in functional impairment. Across trials, the largest and most consistent effects were observed for pain catastrophizing and psychological distress, which is consistent with the cognitive and emotional targets of these interventions, whereas the effects on pain intensity and functional recovery were more modest and heterogeneous. Taken together, these findings support CBT-oriented cognitive-based interventions as important components of multimodal care for chronic musculoskeletal pain, particularly for patients with elevated maladaptive cognitions such as pain catastrophizing, but they should not be regarded as stand-alone treatments capable of fully controlling pain or completely restoring function; rather, they should be integrated into multimodal care pathways aimed at improving pain management and functional capacity. Future high-quality randomized trials with standardized protocols, clearly defined follow-up schedules – for example, common assessments at 3, 6 and 12 months after treatment – and head-to-head comparisons between different CBT-based modalities are needed to clarify which approaches are most effective for which patients.

Looking ahead, there is a growing need for innovative approaches to complement traditional face-to-face CBT in chronic pain management. While CBT is effective, especially in addressing pain catastrophizing, its accessibility is limited for some populations. Digital CBT offers a promising solution by enabling remote treatment, overcoming geographical barriers and providing greater flexibility (Mayhew et al., 2023; Tao et al., 2023). This method could complement traditional CBT by reaching more individuals, particularly those who are unable to attend in-person sessions due to time constraints or other logistical challenges (DeBar et al., 2025). Additionally, adaptive CBT, which tailors interventions to patients’ progress and psychological profiles, could further improve efficacy by providing personalized care (McCracken, 2023; Thorn et al., 2018). From a clinical and service perspective, these findings suggest that CBT and other cognitive-based interventions can be considered as key components of multimodal care for chronic musculoskeletal pain, especially in patients with prominent maladaptive cognitions such as pain catastrophizing. Integrating CBT into routine pain management pathways and expanding delivery through digital platforms or stepped-care models may help to improve access and equity of care. Future studies should focus on comparing these novel CBT modalities with traditional face-to-face interventions, not only in terms of their effectiveness but also regarding cost-effectiveness, patient engagement, and long-term impact on chronic pain outcomes. To optimize these interventions, it is crucial that future research integrates both objective pain measures and self-reported tools to provide a comprehensive understanding of treatment effects. This approach could reduce bias from self-reports and enhance treatment evaluation. By refining CBT delivery through digital platforms and adaptive formats, we can expand access and improve chronic pain management for diverse patient populations.

This article has several limitations related both to the underlying evidence and to our review process. First, only 14 randomized controlled trials with relatively small sample sizes were available, and there was considerable clinical and methodological heterogeneity across trials in terms of intervention content, session number and duration, control conditions, and outcome measures. Although random-effects models and subgroup analyses were used, residual heterogeneity cannot be excluded. Second follow-up schedules and durations varied substantially between studies (see Table 2). To maximise comparability and statistical power, we restricted our quantitative synthesis to post-treatment outcomes, defined as the first assessment after completion of the intervention, and did not conduct separate meta-analyses for predefined short-, medium- and long-term follow-up categories because only a small number of trials contributed data at comparable time windows. Sensitivity analyses that examined study duration as a potential moderator did not materially change the pooled post-treatment effect estimates; however, the variability in follow-up timing and the limited number of long-term assessments mean that our findings provide only limited information about the durability of treatment effects. Third, we restricted inclusion to English-language publications and cannot rule out language or publication bias despite database and reference-list searches. Fourth, we used standardized mean differences for all continuous outcomes to ensure comparability across different instruments, which may reduce the clinical intuitiveness of effect sizes. In the pain meta-analysis, although we extracted only the pain subscales from multidimensional instruments such as AIMS2, IRGL and WOMAC, these measures still differ somewhat in content coverage and recall period from classical unidimensional pain scales (e.g., VAS, NPRS). Pooling them within a single SMD therefore may have introduced conceptual heterogeneity in the pain construct, and this should be borne in mind when interpreting the magnitude of the pooled effect. Fifth, not all included trials evaluated traditional CBT alone; several investigated closely related CBT-based interventions such as PCST, ACT or CFT. This may limit the extent to which the pooled estimates can be generalised to strictly defined CBT protocols, although subgroup analyses suggested broadly similar effects across CBT-based approaches. Finally, this review did not use the GRADE approach to formally evaluate the certainty of evidence, even though GRADE is widely recommended for systematic reviews; this omission represents a methodological limitation and may reduce the strength and interpretability of our conclusions. Future reviews could benefit from incorporating GRADE and more standardised follow-up time points to more comprehensively evaluate the robustness and certainty of the findings.

## 5 Conclusion

This study demonstrated that CBT-oriented cognitive-based interventions grounded in CBT principles produced small-to-moderate improvements in pain intensity and psychological outcomes, and small improvements in functional impairment in adults with chronic musculoskeletal pain. These results support the use of CBT and related CBT-based interventions as a non-pharmacological treatment strategy in clinical practice, rather than as stand-alone treatments. The benefits of these interventions are likely to operate primarily through direct effects on cognitive and emotional processing. By reframing catastrophizing thoughts and regulating negative emotions, CBT-based interventions may help interrupt the vicious cycle of pain, avoidance and functional decline, thereby influencing multiple aspects of the pain experience. Taken together, these findings further emphasize the potential key role of targeted psychological interventions that focus on pain-related cognitive and emotional processing in the management of chronic musculoskeletal pain.

## Data Availability

The original contributions presented in the study are included in the article/Supplementary material, further inquiries can be directed to the corresponding author.
