# Supplementary figures and images for: Efficacy of cognitive behavioral therapy for musculoskeletal pain: a systematic review and meta-analysis
Source: Front Psychol. 2026 Jan 20;16:1705679. doi: 10.3389/fpsyg.2025.1705679 (PMC12864466; doi:10.3389/fpsyg.2025.1705679)

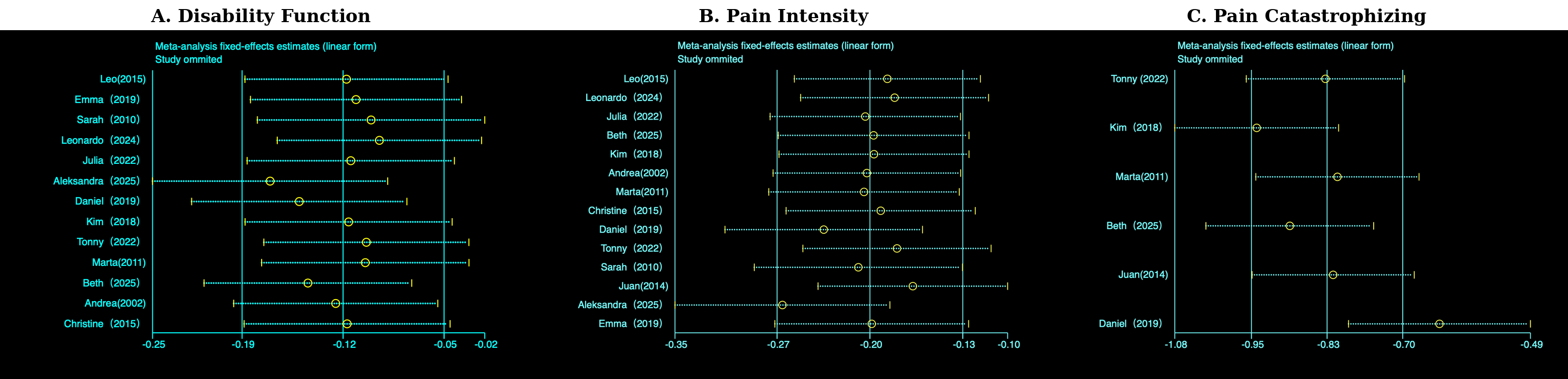

Supplement: Supplementary file 1 [file Image_1.tiff]
